# Multiple Fingerprinting Localization by an Artificial Neural Network

**DOI:** 10.3390/s22197505

**Published:** 2022-10-03

**Authors:** Jaehyun Yoo

**Affiliations:** School of AI Convergence, Sungshin Women’s University, Seoul 02844, Korea; jhyoo@sungshin.ac.kr; Tel.: +82-2-920-7695

**Keywords:** WiFi fingerprinting localization, artificial neural network, multiple targets estimation

## Abstract

Fingerprinting localization is a promising indoor positioning methods thanks to its advantage of using preinstalled infrastructure. For example, WiFi signal strength can be measured by pre-existing WiFi routers. In the offline phase, the fingerprinting localization method first stores of position and RSSI measurement pairs in a dataset. Second, it predicts a target’s location by comparing the stored fingerprint database to the current measurement. The database size is normally huge, and data patterns are complicated; thus, an artificial neural network is used to model the relationship of fingerprints and locations. The existing fingerprinting locations, however, have been developed to predict only single locations. In practice, many users may require positioning services, and as such, the core algorithm should be capable of multiple localizations, which is the main contribution of this paper. In this paper, multiple fingerprinting localization is developed based on an artificial neural network and an analysis of the number of targets that can be estimated without loss of accuracy is conducted by experiments.

## 1. Introduction

**Figure 1:**
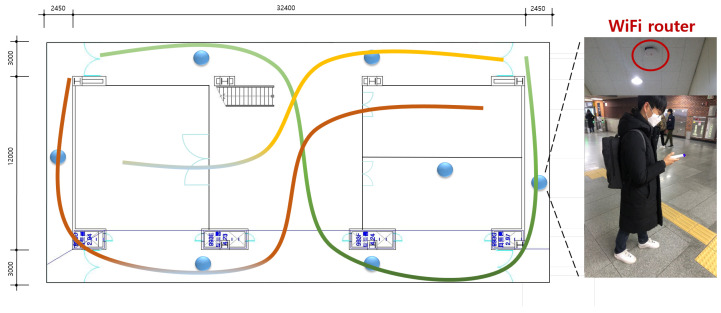
Multiple fingerprinting indoor localization.

There are different methods for indoor localization based on RF signals. In the Time of Arrival (TOA), Time Difference of Arrival (TDOA), and Angle of Arrival (AoA) methods [1], a target is estimated by calculating the time differences, distances, and angles, respectively, between a target and transmitters using the received signals from deployed beacons. On the other hand, the WiFi fingerprinting approach estimates a target location based on pattern recognition through machine learning methods. This paper focuses on WiFi fingerprinting localization. Figure 1 illustrates multiple fingerprinting indoor localization using a WiFi router and its measurements, with different colors representing different users’ trajectories.

The Wi-Fi indoor positioning method requires a database of signal strength fingerprints collected from surrounding areas for each predefined location. In an indoor environment with many obstacles, such as walls blocking radio waves, more fingerprint data are required for higher positioning accuracy. In considering the resolution of the Wi-Fi signal strength according to the distance, fingerprint data are usually collected at intervals of 1 to 2 m to form a training database. When providing the positioning service, the current location is predicted by comparing the currently received signal strength set with the previously built database. Because this is the same as the training and test phases of machine learning approaches, *k*-nearest neighbors, support vector machine, Gaussian processes, and deep neural networks can be used for fingerprinting localization [2,3,4,5].

When applying a deep neural network for WiFi indoor positioning, a wireless signal strength set is configured as an input of the neural network, a location is set as an output terminal, then a hidden network is learned as a positioning model. In the learning phase, the model is trained using the signal strength fingerprint data collected over the entire positioning region, and in the test phase the predicted position is obtained by inputting the query signal strength set to the learned neural network model.

Most indoor localization focuses on estimating the location of a single target [6,7,8,9,10,11,12], with only a few addressing multi-target localization. In [13,14], multi-targeting was achieved using UHF RFID and microphone devices, respectively. These methods require a number of devices to be located in a room, which is not feasible for general localization purposes. In [15,16], a WiFi fingerprint was used for multiple targets. In [15], an extraction method of signal reflection corresponding to multiple users was proposed. This technique requires a controlled environment in which measurements of near-static WiFi devices are available. In [16], graph-based optimization was used to fuse WiFi and dead reckoning for a single target. When this filtration method is expanded to multi-user scenarios, the number of users and measurements strongly affects the time complexity.

Existing neural network-based positioning technology is inefficient in terms of real-time processing speed because it has no choice but to perform estimation calculations as many times as requested when a large number of users request positioning service. When indoor positioning service is provided from a server to local smart-devices in a centralized way, the service quality depends on the time required to estimate the location, not on the learning processing time. Therefore, a new positioning method for simultaneous multiple users is needed.

As it becomes necessary to infer many locations at the same time, the dimensionality of the machine learning input data increases. Because a deep neural network has excellent reasoning ability for learning high-dimensional data, it is suitable for multi-location estimation problems. This paper handles prepocessing and standardization of WiFi received signal strength indicator (RSSI) measurements, which are significant for obtaining good learning performance.

To evaluate the proposed multi-target estimation approach based on an artificial neural network for predicting 3D information, including both location and floor, experimental data were collected in a five-story building. We analyzed the number of targets that could be estimated without loss of significant accuracy. The experimental results show that fifteen targets can be accurately estimated with similar performance in the single localization.

The rest of this paper is organized as follows. Section 2 describes the proposed multi-target WiFi fingerprinting localization approach. Section 3 reports our experimental results. Section 4 presents our conclusions.

## 2. WiFi Fingerprinting Indoor Localization

Suppose that there are a total of *d* WiFi routers in which the indoor positioning service is to be established. In a multi-story building, the location is expressed as a three-dimensional vector such as (*x* coordinate, *y* coordinate, floor). By storing RSSI measurements received from *d* routers at collecting locations, a fingerprint database consisting of (location, *d*-dimensional RSSI set) pairs is constructed, in which router locations are not necessarily known. To include environmental variations in the data, RSSIs are measured several times at a location. Table 1 shows the fingerprint database format.

Because certain signals from distantly located routers are not observable, the fingerprint database has many elements filled with empty spaces (null). Because null spaces cannot be processed in machine learning, these are intentionally converted to a −100 dB value.

The following Section 2.1 and Section 2.2 present feature extraction for WiFi RSSI measurements and standardization for labelled location data, respectively. Section 2.3 presents deep learning-based multi-target localization.

### 2.1. WiFi RSSI Feature Extraction

Principal component analysis (PCA) [17] is a data transformation method for reducing the dimensionality of data and extracting the features data by minimizing information loss. It can be understood as an unsupervised learning method, as it does not require label information such as location; thus, in this paper, it is used to extract the features of WiFi fingerprint measurement data.

Suppose $D={\left\{\left(x_{i},y_{i}\right)\right\}}_{i=1}^{N}$ are fingerprint training datasets, where $x_{i}$ is a vector of RSSI measurements at a location $y_{i}$ provided by

(1) $$\begin{array}{cc} x_{i} & ={\left[s_{i}^{1},s_{i}^{2},\cdots ,s_{i}^{d}\right]}^{T}\in R^{d}, \end{array}$$

(2) $$\begin{array}{cc} y_{i} & ={\left[x_{i},y_{i},f_{i}\right]}^{T}\in R^{3}, \end{array}$$

where $s_{i}^{j}$ is a scalar indicating the RSSI value at the *i*th location received from the *j*th WiFi router over a total of *d* routers, $x_{i}$ and $y_{i}$ are the 2D location, and $f_{i}$ is floor level.

The original data *x* are transformed to the feature dataset $\bar{x}$ by the transformation matrix *Q*, as follows:

(3) $$\bar{x}\leftarrow Q\cdot x,$$

(4) $$\begin{array}{c} \mathrm{where}\;Q\in R^{l\times d},\;\bar{x}\in R^{l},x\in R^{d},\;l\ll d. \end{array}$$

In this paper, finding an optimal *Q* is achieved by the PCA algorithm, which solves a generalized eigenvalue problem. Let ${\left\{\rho_{i}\right\}}_{i=1}^{d}$ be the generalized eigenvectors associated with the generalized eigenvalues ${\left\{\lambda_{i}\right\}}_{i=1}^{d}$ of the following generalized eigenvalue problem:

(5) $$\begin{array}{c} A\rho_{i}=\lambda_{i}I\rho_{i},\;\;\;i=1,\ldots ,d, \end{array}$$

where *I* is an identity matrix and *A* is a scatter matrix provided by

(6) $$\begin{array}{c} A=\sum_{i=1}^{N}\left(x_{i}-\mu \right){\left(x_{i}-\mu \right)}^{T}, \end{array}$$

with the mean of all the RSSI samples μ:

(7) $$\begin{array}{c} \mu =\frac{1}{N}\sum_{i=1}^{N}x_{i}. \end{array}$$

Then, the optimal matrix $\hat{Q}$ is calculated by solving the optimization problem presented by

(8) $$\begin{array}{c} \hat{Q}={\mathrm{argmax}}_{Q}\frac{|Q^{T}AQ|}{|Q^{T}IQ|}, \end{array}$$

where the condition of the eigenvalue problem is that the generalized eigenvectors are orthogonal:

(9) $$\begin{array}{c} \rho_{i}^{T}\rho_{j}=0,\;\;\;\mathrm{for}\;i\neq j, \end{array}$$

and the generalized eigenvectors are normalized:

(10) $$\begin{array}{c} \rho_{i}^{T}\rho_{i}=1,\;\;\;\mathrm{for}\;i=1,\ldots ,d. \end{array}$$

Finally, when the eigenvalues are sorted in descending order such that

(11) $$\begin{array}{c} \lambda_{1}\geq \lambda_{2}\geq \cdots \geq \lambda_{d}, \end{array}$$

the transformation matrix *Q* is provided by

(12) $$\begin{array}{c} Q=\left(\sqrt{\lambda_{1}}\rho_{1}|\sqrt{\lambda_{2}}\rho_{2}\left|\cdots \right|\sqrt{\lambda_{l}}\rho_{l}\right)\in R^{l\times d}. \end{array}$$

It should be noted that the last small values of the eigenvalues and eigenvectors do not affect the transformation. Thus, we choose a proper *l* to cut down the original dimensionality from *d* to *l*.

### 2.2. Label Standardization

In a WiFi fingerprint localization system, the two-dimensional coordinate values in the position vector $y_{i}$ are generally expressed in meters and the floor values are expressed as integers, ensuring that the scale of the data values between position and floor is different. In addition, the location data are not evenly distributed, as the two-dimensional location is concentrated in the hallway due to the nature of the indoor space. This characteristic can have a negative effect on the accuracy of all general machine learning algorithms, including artificial neural networks. In this paper, standardization is used to convert the original location label data $y_{i}={\left[x_{i},y_{i},f_{i}\right]}^{T}$ to ${\bar{y}}_{i}$, as follows:

(13) $$\begin{array}{c} {\bar{y}}_{i}\leftarrow \left(y_{i}-\mu_{s}\right)/\sigma_{s}, \end{array}$$

where $\mu_{s}$ and $\sigma_{s}$ are the mean and standard deviation of ${\left\{y_{i}\right\}}_{i=1}^{N}$, respectively.

As a summary of the data preprecessing, the PCA transforms the RSSI measurement data from *x* to $\bar{x}$, and standardization transforms the location data from *y* to $\bar{y}$. As a result, the new feature database $\bar{D}={\left\{{\bar{x}}_{i},{\bar{y}}_{i}\right\}}_{i=1}^{N}$ is obtained.

### 2.3. Multi-Target Estimation Based on Artificial Neural Network

An artificial neural network consists of several layers. In a layer, neural nodes have a linear combination composed of learning parameters. When the combined signal propagates to the next layer, a nonlinear model such as a rectified linear unit (ReLU) activation function decides the output of the layer. As the signal propagates to multiple layers, the nonlinearity increases. The success of deep learning in finding optimal node parameters depends on determining the appropriate number of hidden layers and neural nodes.

**Figure 2:**
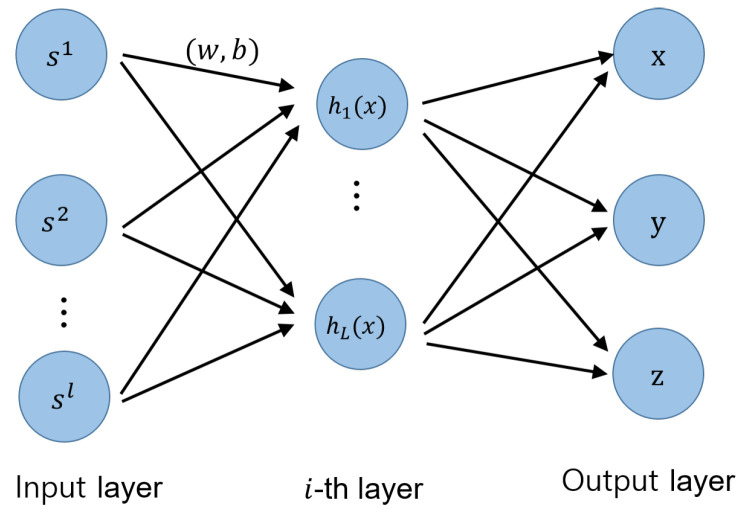
Deep neural network structure for single-position learning.

Figure 2 shows the structure of a deep neural network for general single-location learning. In this case, the input layer of the neural network uses the *l*-dimensional RSSI vector, $\bar{x}\in R^{l}$ in (3).

**Figure 3:**
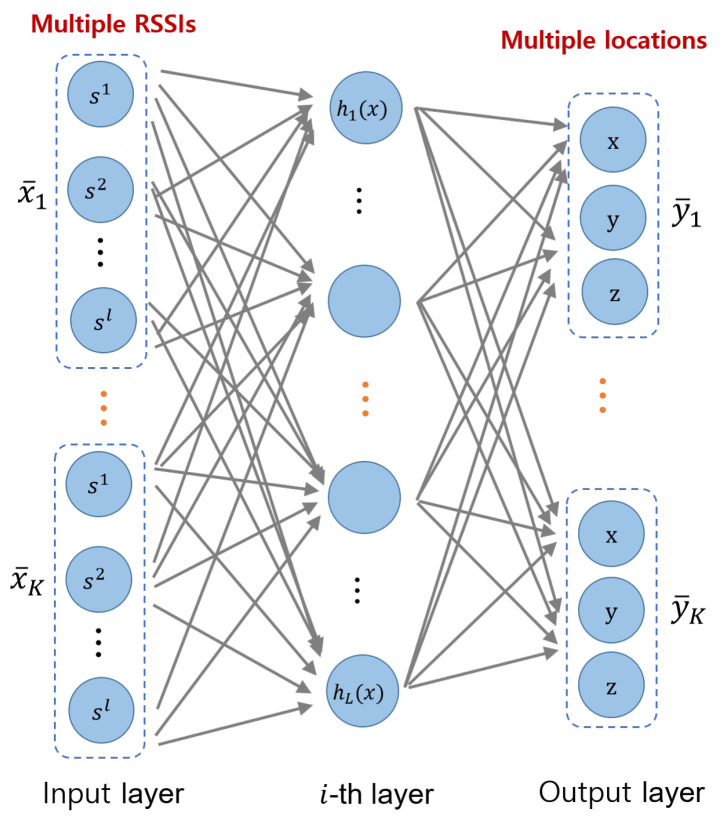
Extended deep neural network structure for multi-position learning.

Figure 3 is a neural network structure for multi-location recognition. It is a parallel extension of the single-location neural network shown in Figure 2. As shown in Figure 3, as the number of positions to be simultaneously estimated increases, more neural network nodes and a higher number of learning parameters are required. When the input data and output data are expanded, as shown in Figure 3, the dimensionality of the data increases.

General machine learning techniques including deep neural network require large amounts of training data for high dimensionality data [18]. The datasets $\bar{D}={\left\{{\bar{x}}_{i},{\bar{y}}_{i}\right\}}_{i=1}^{N}$ have undergone preprocessing for single positioning. Given a fixed amount of data $\bar{D}$, in order to generate sufficient data for multi-location learning (that is, to make up the input/output dataset represented in Figure 3), an additional rendering operation is required.

Let us suppose that the number of multiple locations to be estimated is *K*; then, the composition of input data and output data of the neural network is as follows:  

(14) $$\begin{array}{cc} X_{i} & ={\left[{\bar{x}}_{1}^{T},{\bar{x}}_{2}^{T},\cdots ,{\bar{x}}_{K}^{T}\right]}^{T}, \end{array}$$

(15) $$\begin{array}{cc} Y_{i} & ={\left[{\bar{y}}_{1}^{T},{\bar{y}}_{2}^{T},\cdots ,{\bar{y}}_{K}^{T}\right]}^{T}. \end{array}$$

In (14) and (15), the *j*th component ${\bar{x}}_{j}^{T}$ and ${\bar{y}}_{j}^{T}$ are sampled from the original $\bar{D}={\left\{{\bar{x}}_{i},{\bar{y}}_{i}\right\}}_{i=1}^{N}$. It should be noted here that according to the sorting order of the pairs $({\bar{x}}_{j}^{T},{\bar{y}}_{j}^{T})\;\forall j=1,\cdots ,K$, data $X_{i}$ and $Y_{i}$ can be heterogeneous. For example, $X_{1}={\left[{\bar{x}}_{1}^{T},{\bar{x}}_{2}^{T},\cdots ,{\bar{x}}_{K}^{T}\right]}^{T}$ and $X_{2}={\left[{\bar{x}}_{K}^{T},{\bar{x}}_{K-1}^{T},\cdots ,{\bar{x}}_{1}^{T}\right]}^{T}$ are different sets unless all elements are equal. Therefore, an appropriate amount of new training data to be used for multi-location learning can be generated by repeating and aligning random extractions at $\bar{D}$ while keeping the condition of ${\bar{y}}_{j}^{T}\neq {\bar{y}}_{k}^{T}$ for any j≠k∈{1,⋯,N}. As a result, new training datasets for the multiple targets $F={\left\{X_{i},Y_{i}\right\}}_{i=1}^{c}$ are made, in which parameter *c* is defined by a developer. Algorithm 1 summarizes the algorithm used to produce multi-target training data *F* from $\bar{D}$.
**Algorithm 1** Rendering multi-target fingerprint training dataset**Input:**$\bar{D}={\left\{{\bar{x}}_{i},{\bar{y}}_{i}\right\}}_{i=1}^{N}$, single target fingerprint training data
**Output:**$F={\left\{X_{i},Y_{i}\right\}}_{i=1}^{c}$, multi-target fingerprint training data
**Initialization:**
Set the desired number of multi-target training data *c* and the multi-target *K*, and define $X_{i}=Y_{i}=\phi$ as empty sets.  
1:Initialize generator with $\theta_{G}$, discriminator with $\theta_{D}$ and classifier with $\theta_{C}$.2:**for**i=1,2,…,c**do**3:    **for** j=1,2,…,K **do**4:   Sample $\left({\bar{x}}_{j},{\bar{y}}_{j}\right)$ randomly from $\bar{D}$ subject to ${\bar{x}}_{j}\notin X_{i}$ and ${\bar{y}}_{j}\notin Y_{i}$.5:        Add the samples by $X_{i}\leftarrow X_{i}\cup {\bar{x}}_{j}$ and $Y_{i}\leftarrow Y_{i}\cup {\bar{y}}_{j}$6:    **end for**7:    (Here, the *i*th data in Equations (14) and (15) are made up.)8:**end for**


## 3. Experimental Results

### 3.1. Setup

**Figure 4:**
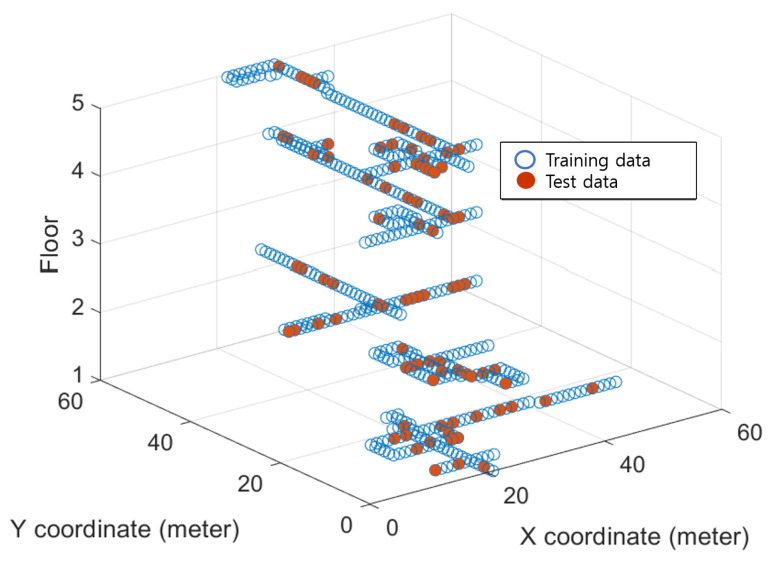
Experimental WiFi fingerprint data distribution along hallway of a multi-story building.

Figure 4 shows the experimental five-story building with a size of 60 × 60 m used to evaluate the developed localization. For Wi-Fi fingerprint data collection, RSSI measurements and the corresponding location data were collected along the corridor at intervals of 1.5 m, and a total of 2207 learning data and 100 test data measurements were collected distributed among the five floors. The RSSI data dimensionality is determined by the number of routers *d*, the total number of routers found in this test building is d=508, and the amount of data is N=2207. The 2D location data were processed according to the Universal Transverse Mercator coordinate system, and the floor values are integers from 1 to 5.

The deep neural network was trained and tested using Tensorflow. The training parameters of the neural network were initialized through the Xavier method, the activation function was defined as the ReLU function, the gradient descent method was performed through the Adam-Optimizer, and the loss function of the training cost function was defined as the root mean square error.

Through feature extraction using PCA, the original data with d=508 were reduced to feature data with l=14. The reason for this setup is that the eigenvalues of the transformed feature data after the fourteenth dimension of the PCA-driven feature data are relatively small.

### 3.2. Result

The structure of the proposed deep neural network for indoor positioning consists of one hidden layer and 220 nodes. This setup was used because when the number of hidden layers was set two or more, the learning performance was degraded.

**Figure 5:**
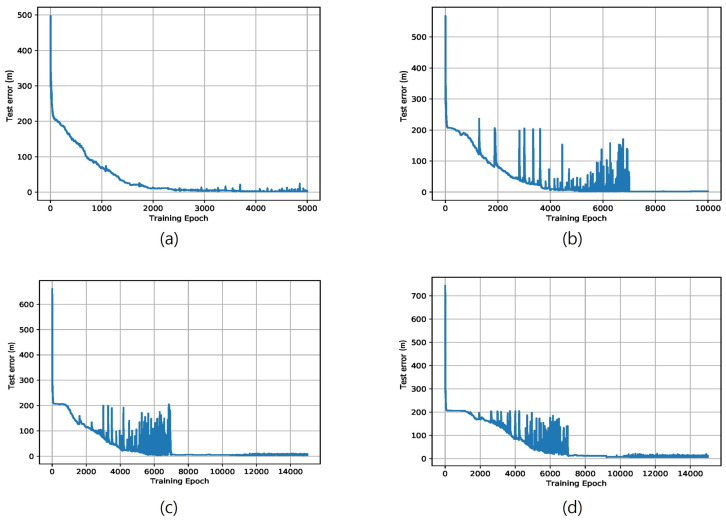
Positioning test error by deep neural network according to the number of estimated positions: (**a**) K=10, (**b**) K=15, (**c**) K=20, (**d**) K=25.

Figure 5 shows the test performance according to the training epoch when the number of positions to be estimated is 10, 15, 20, and 25, respectively. The test error on the *y*-axis is defined as the sum of the Euclidean distance on the two-dimensional coordinates and the 1 m penalty for the three-dimensional coordinates (i.e., floor), and is calculated as the average of 100K number of measurements. Because the output value of the deep network is not limited to within the real space, however, the test error may be very large in the early stages of learning. As shown in Figure 5, over all training results, the test error decreases according to the increment of the training epoch. However, as the number of simultaneous estimated positions increases, the converged test mean error increases.

Sometimes, a high test error occurs intermittently during the learning optimization process, which shows the sensitivity of the multi-position neural network model to changes in the neural network’s learning parameters.

As the size of the learning rate increases during the learning process, the change rate of the updated parameter values is large. In general, a relatively large learning rate is set in the early stages of learning, and a small rate is set in the later stages to guarantee learning convergence. For our experiment, convergence was secured by changing the learning rate from 0.01 to 0.001 after around 7000 epochs, meaning that the test error plots after 7000 epochs are almost changeless, as in Figure 5.

Table 2 shows the number of training epochs, the duration of training, the number of training data items, and the average error. The mean error/standard deviation error indicated in the fifth column of Table 2 is the average of only the test errors from the last epoch of the learning phase to the 100th time. According to the number of estimated positions increasing or decreasing, the number of training epochs, the training duration, and the amount of data all increase or decrease. Theis result is obvious in that when the number of multi-targets *K* increases, the average error increases. In this experiment, when the number of position estimates was K=10 and K=15, good performance was obtained without much difference from single position estimation accuracy. When K>15, however, the estimation performance becomes too inaccurate.

As a baseline to compare accuracy and test time, *k*-NN [19] is implemented, in which k=10 after fine tuning; this approach uses the same PCA-driven feature data as the input. For a single target, the *k*-NN experiment achieved an error of 2.69 m, which is similar to the performance of proposed method when the target number was 15. The *k*-NN took 70 ms for test time. Because the test-time complexity of *k*-NN is O(nm), in which *n* and *m* are the number of training and test samples, respectively, it consumes time in proportional to increment of the number of targets. On the other hand, the proposed algorithm took 50∼55 ms regardless of variations in the number of targets because of the consistent neural network structure.

## 4. Conclusions

In this paper, WiFi-based indoor positioning using a deep neural network for multi-location learning and estimation is proposed. WiFi wireless signal strength fingerprint original data were pre-processed using PCA to extract feature data and location through standardization for use as input data for a neural network. The input data were further rendered for multi-location learning in an artificial neural network structure. A positioning experiment performed in a five-story building confirmed that accurate learning results were derived when estimating less than 20 locations at the same time.

## Figures and Tables

**Table 1:** Example of WiFi RSSI fingerprint database.

	AP 1	AP 2	⋯	AP *d*
Position 1	−85 dBm	−68 dBm	⋯	Null
Position 2	Null	−51 dBm	⋯	−92 dBm
⋮	⋮	⋮	⋮	⋮
Position *N*	Null	Null	⋯	−45 dBm

**Table 2:** Experimental results.

Positions *K*	Learning Iterations	Learning Time (s)	# of Training DataData	Ave. Test Error ± Std (m)
1	3500	170.49	2575	2.29 ± 0.002
10	5000	394.43	67,080	2.60 ± 1.55
15	10,000	994.16	67,080	2.68 ± 0.03
20	15,000	1818.18	77,400	6.07 ± 1.48
25	15,000	2474.04	103,200	9.93 ± 2.86

## Data Availability

Not applicable.
